# Immigration Policy Vulnerability Linked to Adverse Mental Health Among Latino Day Laborers

**DOI:** 10.1007/s10903-021-01254-z

**Published:** 2021-08-28

**Authors:** Alein Y. Haro-Ramos, Hector P. Rodriguez

**Affiliations:** School of Public Health, University of California, Berkeley, 2121 Berkeley Way #5427, Berkeley, CA 94704 USA

**Keywords:** Mental health, Immigration policy, Day laborers, Stress, Legal vulnerability, Undocumented

## Abstract

Latino day laborers in the United States are socially and economically vulnerable due to exclusionary immigration policies. Using data from a multi-mode survey, we examine the relationship between immigration policy legal vulnerability and mental health outcomes among 138 Latino, male day laborers (mean age = 45.65, SD = 12.05). Multivariable linear and logistic regression models separately estimated the effect of legal vulnerability, as measured by the Perceived Immigration Policy Effects Scale, on anxiety and depression symptoms and a positive depression and anxiety screening, respectively. Approximately 26.1% and 27.9% of day laborers reported depression and anxiety symptoms, respectively. In each adjusted model, we find a positive relationship between legal vulnerability and adverse mental health. Immigration policy legal vulnerability is associated with more depression and anxiety symptoms among Latino day laborers. Policies to reduce legal vulnerability, such as pathways to citizenship and employment authorization, may support Latino day laborers' mental health outcomes.

## Introduction

Latino day laborers (LDLs) are a subgroup of the 11 million undocumented individuals [1] in the United States (U.S.) who are structurally vulnerable due to their legal status and a clustering of risk factors beyond their direct control [2]. The social conditions of day laborers —who are primarily undocumented men from Mexico and Central America—are shaped by structural forces in the form of exclusionary policies that racialize and criminalize immigrants [3–6]. Since 1996 U.S. laws have helped cement the association between undocumented immigrants and criminality [6], and this criminalization has negative health consequences for immigrants, their families, and communities [7, 8]. Furthermore, anti-immigrant rhetoric and draconian U.S. policy toward immigrants escalated during the Trump Administration [9]. Some recent immigration policy changes include increased enforcement [10], barriers to legal migration [11], and the separation of migrant children from their parents [12]. Current U.S. immigration policies shape the everyday work and life experiences of immigrants. As such, self-perceived immigration policy vulnerability, including social exclusion, discrimination, and fear of family separation, is a critical indicator of immigration policies’ effects on immigrants’ lives. While several studies have identified a relationship between legal status and mental health, few studies have operationalized legal vulnerability as a multidimensional construct. Immigration policy vulnerability may result in adverse mental health outcomes in the immigrant community by increasing exposure to chronic psychosocial stressors and creating a hostile environment.

Immigration policy is a structural determinant of health for day laborers because it shapes social and economic conditions. Immigration policies also determine immigrants’ access to salubrious resources, risk of deportation, and employment opportunities based on legal status [13]. For example, the 1986 Immigration Reform and Control Act (IRCA) makes it illegal to hire undocumented people [14]. The exclusion of workers based on legal status impedes economic and social integration and leads to undocumented workers’ segmentation in low-paying and high-risk jobs [15, 16]. Undocumented immigrants may rely on the informal day labor market where economic opportunities are limited [16] and occupational risks are abundant [17, 18]. Therefore, exclusionary immigration policies can be detrimental to day laborers' health by shaping social determinants of health-related to economic and employment stability, community and social context, and access to health care services [19–21].

Previous studies have attributed disparities in mental health among immigrant populations due to acculturative stress [22]. A more recent line of qualitative work has focused on immigrants’ experiences of discrimination in anti-immigrant contexts, focusing on policies such as Arizona’s SB 1070, which allowed local law enforcement officers to consider phenotypic characteristics or national origin to request proof of immigration status [19, 21, 23]. The present work builds on the existing evidence and centers on legal vulnerability associated with exclusionary immigration policies and its relationship to immigrants’ mental health outcomes.

## Conceptual Framework

We use the Minority Stress Framework (MSF) to inform our inquiry. The MSF perspective underscores the cumulative effects of social stressors on adverse health outcomes among marginalized groups [24]. For day laborers, punitive immigration policies, increased enforcement actions, and negative stigmatization of immigrants increase susceptibility to adverse mental health outcomes. These factors also shape access to psychosocial and institutional resources that can be used to mitigate the effects of discrimination and anticipatory threats [25, 26]. In sum, adverse mental health results from greater exposure to stressors caused by social systems that structurally discriminate against minority populations [27].

Immigration policies can affect immigrants’ mental health and well-being through direct and indirect mechanisms [28]. Directly, policies can enhance or restrict access to health-related public benefits such as food assistance programs and health insurance [29]. For example, federally funded public programs generally exclude undocumented immigrants [30, 31], and recent regulatory changes to the Public Charge rule discourage immigrants from using public benefits due to fear of perceived immigration consequences [32]. Indirectly, immigration policies can operate through psychosocial mechanisms by creating a climate of fear and instability. Increased interior immigration enforcement targets all immigrants regardless of their criminal background, and, since 1996, new immigration policies have been enacted to identify, apprehend, detain, and ultimately deport all undocumented immigrants [7, 8, 10]. An exclusionary policy climate can cause fear and discourage immigrants from engaging in many aspects of life.

The psychological distress caused by discrimination, the effortful coping with anticipatory threats of family separation, and social exclusion limiting access to resources all impact well-being. Among Latino male day laborers, correlates of adverse mental health include being homeless, experiencing discrimination, higher levels of acculturation stress, and being single [4]. These social stressors may lead to the overstimulation of the stress-response system, which can increase disease vulnerability and risk of adverse physical and mental health [33, 34]. An environment of fear, distrust, and perception of surveillance increases stress levels and promotes unhealthy behavioral coping strategies. For example, in a hostile environment, immigrants may socially isolate and avoid public spaces, health care institutions, and government offices [8, 35, 36]. Preventing exposure to threatening situations, such as deportation, translates to immigrants being disadvantaged in vital social determinants of health. Overall, a hostile environment is associated with poor mental health outcomes among foreign-born Latinos [23, 37, 38]. The ever-present uncertainty and instability produced by current immigration policies influence day laborers’ health-seeking behaviors [39], livelihood and wages [14], vulnerability in the streets [17], and their chances in the informal and formal labor market [40]. The daily manifestations of immigration policy vulnerability have the potential to affect day laborers’ mental health outcomes. We hypothesize that higher perceived immigration policy vulnerability among day laborers will be associated with more depression and anxiety symptoms and a greater risk of screening positive for clinical depression and anxiety.

## Methods

### Study Sample

From February to July 2020, we recruited 138 eligible immigrant Latino male day laborers using an assistant administered, multi-mode survey. Eligibility criteria included being 18 years of age or older and having performed day labor work in the past three months. We define day labor as temporary and flexible work obtained in the informal market; participants could be hired across numerous industries (i.e., construction, landscape, farm work). We used a 60-min structured interview of 110 previously validated items in which interviewers read the survey items to the participants and recorded their responses. Participants had the option of responding to the questions in Spanish or English, but all opted for Spanish. Each participant received a $20 compensation. Common reasons for refusals to participate included the lack of time and the prioritization of seeking employment opportunities. Participants could leave at any time during the interview and skip over any questions.

### Data Collection

We recruited and administered the survey in person or over the telephone before the California shelter-in-place ordinance began on March 19, 2020, due to COVID-19. After that, we collected information from participants solely by telephone. We used a census list of day laborers enrolled in our community partner’s Day Laborer Program (DLP) in the East San Francisco Bay Area. The DLP is a job placement assistance program that helps economically disadvantaged migrants acquire educational, vocational, and social skills to build self-sufficiency. The community partner conducts community outreach where day laborers congregate to inform them of the DLP and other services. Researchers called every member (374 individuals) of the DLP census list to recruit participants. Fifty individuals in the census list were not reached due to the listing of an invalid phone number. Among the 324 accessible individuals through the DLP, the response rate was 22.8% (n = 74/324), and the completion rate was 95.9% (n = 71/74). We supplemented our recruitment at public hiring sites in the neighboring area (≤ 5 miles) of the community partners’ headquarters. An additional 64 respondents were interviewed at hiring sites; the completion rate for this subsample was 82.8% (n = 53/64). We used two distinct recruitment and survey administration modes to increase response rates and yield a larger sample. The Committee for Protection of Human Subjects at the University of California, Berkeley approved this study (Protocol IRB-12499).

### Measures

#### Independent Variable

Our independent variable of interest was legal vulnerability experiences attributed to immigration policy in the US. Immigration policy vulnerabilities were assessed using the Perceived Immigration Policy Effects Scale (PIPES) instrument, which has been validated among Spanish-speaking migrants [41]. The instrument was initially used among Latino immigrant parents [41] and since then has been used with other populations, including US-born Latino adolescents [9] and Mexican mothers in a farmworker community [42]. PIPES captures discrimination, social exclusion, and the threat of family separation attributed to immigration policies using 17 items scored on a 5-point Likert scale from never to always (α = 0.90). Items were summed to obtain a score that ranged from 17 to 85, with each question scored from 1 (“Never”) to 5 (“Always”). This instrument explicitly accounts for the perceived effects of immigration policy vis-à-vis immigrants’ interactions (or lack thereof) with mainstream society, experiences of discrimination, and fears of family separation. Higher scores on the overall measure indicate a higher level of legal vulnerability.

#### Outcome Variables

The two study outcome variables are depression and anxiety. Day laborers’ depressive symptoms were assessed using the Patient-Health-Questionnaire 8 (PHQ-8), which has been used and validated among Latinos and Spanish-speaking patients in clinical and community settings [4, 43]. We opted for the PHQ-8 instead of the PHQ-9, which assesses suicidal ideation, due to participant safety concerns; we did not want to trigger a negative reaction without providing participants with adequate risk management support. Furthermore, a meta-analysis on the equivalency of the PHQ-8 and PHQ-9 found the measures were highly correlated (*r* = 0.996) [44], and the PHQ-8 is no less useful than the PHQ-9 in screening for a depressive disorder [45]. The PHQ-8 measures depression with eight questions (“Over the last 2 weeks, how often have you been bothered by any of the following problems? Little interest or pleasure in doing things,” “Feeling down, depressed, or hopeless”) scored on a 4-point Likert scale from not at all to nearly every day (α = 0.76). Items are summed to obtain a total score ranging from 0 to 24, with each question scored from 0 (“Not at all”) to 3 (“Nearly every day”). The cutoff for a positive PHQ-8 screening is a score of ≥ 5 for at least mild depression.

The Generalized Anxiety Disorder 7 scale (GAD-7) measures generalized anxiety symptoms using seven items (“Over the last two weeks, how often have you been bothered by the following problems? Feeling nervous, anxious, or on edge,” “Not being able to stop or control worrying”) scored on a 4-point Likert scale from not at all to nearly every day (α = 0.75) [46]. Items are summed to obtain a total score ranging from 0 to 21, with each question scored from 0 (“Not at all”) to 3 (“Nearly every day”). The GAD-7 has also been validated with Spanish-speaking Latinos in the U.S. [47]. A score of ≥ 5 yields a positive GAD-7 screening for at least mild generalized anxiety disorder.

#### Control Variables

Covariates hypothesized to be predictors of anxiety and depression included age, education, and country of origin. Other confounders of the immigration policy legal vulnerability and mental health relationship include years in the U.S., marital/cohabitation status (0 = single, 1 = married but spouse lives abroad, 2 = married/cohabiting), English fluency (0 = none*,* 1 = a little, 2 = get by, and 3 = well), native language (Spanish vs. indigenous), and average weekly earnings. We also included an indicator for whether the survey took place after the statewide shelter-in-place ordinance went into effect in California as social distancing during the COVID-19 pandemic may be associated with social isolation and depression.

### Analysis

Descriptive statistics were conducted to compare day laborers’ demographic and clinical characteristics overall and by their PIPES score categories. In the descriptive analysis, we dichotomized total PIPES scores into low (a mean response of *never* or *rarely* on all items; a score of 17—34) or high (a mean response of *sometimes* and *above*; a score of 35 +). T-tests were used for continuous variables and Chi-square tests for categorical variables. We stratified the average PIPES score, PHQ-8 and GAD-7 scores, and PHQ-8 and GAD-7 screening classifications by low and high PIPES categories. We describe the distribution of responses to each of the 17 PIPES scale items to understand how immigration policy manifests in immigrant day laborers’ lives.

While in the descriptive analysis we dichotomized PIPES scores for comparative purposes, in all regression analyses we use a continuous and standardized measure of the PIPES score. To check for multicollinearity, we calculated the variance inflation factor (VIF) scores for all coefficients in the regression models (age, education, marital status, country of birth, native language, English fluency, average weekly earnings, and an indicator of whether the survey took place after the California shelter in place ordinance was enacted). Years in the U.S. was excluded from the final models due to its high correlation with age. We used multivariable linear regression in Models 1 and 2 to estimate the relationship between PHQ-8 and GAD-7 scores, respectively, and standardized PIPES scores, adjusting for all covariates. In Models 3 and 4, we used multivariable logistic regression for positive depression and anxiety screenings, respectively, as a function of standardized PIPES scores and all covariates previously mentioned. All statistical analyses were completed using STATA 15.0.

## Results

Of the 138 participants interviewed, 14 were excluded for missing outcome data. Among the 124 valid cases included in the analysis, the distribution of demographic characteristics is comparable for respondents with high and low PIPES scores, with no significant differences in duration in the U.S., weekly income, primary language, marital status, age, and English fluency (Table 1). Respondents with high PIPES scores were more likely to have lower educational attainment (5 versus 7 years, p = 0.006) relative to those with low PIPES scores. The average age of respondents was 45.65 (SD = 12.05, range: 19 to 66) years, and they had an average of 6.34 (SD = 3.76, range: 0 to 14) years of education and had been in the U.S. for an average of 16.60 (SD = 10.10, range: 1 to 40) years. Over half (54.0%) of respondents were from Guatemala, 33.1% were from Mexico, and 12.9% were from El Salvador or Honduras. One in three respondents reported weekly earnings of less than $300, and 40% reported earning $301 to $600 per week. Dollar values are expressed as 2020 dollars. Roughly 8 in 10 respondents reported Spanish as their primary language, and the remaining 20% reported speaking an indigenous dialect such as Mam, Quiche, and Jakaltec. Almost half of our sample reported having no spouse, while 19% indicated having a partner living abroad, and 33% lived with their partner. Almost 1 in 5 respondents said they speak no English.

**Table 1:** Day laborer characteristics for the overall EBDLS sample and compared between low and high PIPES Scores, 2020, (N = 124)

Variable	Total	Low PIPES^a^	High PIPES^b^	p-value
	N = 124	N = 78	N = 46	
Age, mean (SD)	45.65 (12.05)	47.04 (11.34)	43.30 (12.95)	0.096
Duration in the US, mean (SD)	16.60 (10.10)	17.38 (10.62)	15.28 (9.10)	0.26
Education, mean (SD)	6.34 (3.76)	7.05 (3.67)	5.13 (3.63)	0.006
After shelter in place, % (n)	56.45% (70)	55.13% (43)	58.70% (27)	0.70
Country of birth, % (n)				0.078
Mexico	33.06% (41)	34.62% (27)	30.43% (14)	
Guatemala	54.03% (67)	57.69% (45)	47.83% (22)	
El Salvador or Honduras	12.90% (16)	7.69% (6)	21.74% (10)	
Weekly income, % (n)				0.38
$0–$300	35.48% (44)	33.33% (26)	39.13% (18)	
$301–$600	38.71% (48)	37.18% (29)	41.30% (19)	
$601–$1000	18.55% (23)	19.23% (15)	17.39% (8)	
$1000 +	7.26% (9)	10.26% (8)	2.17% (1)	
Primary language, % (n)				0.67
Spanish	80.65% (100)	79.49% (62)	82.61% (38)	
Indigenous	19.35% (24)	20.51% (16)	17.39% (8)	
Marital status, % (n)				0.38
No spouse	48.39% (60)	52.56% (41)	41.30% (19)	
Has partner & live separately	18.55% (23)	15.38% (12)	23.91% (11)	
Partner & living together	33.06% (41)	32.05% (25)	34.78% (16)	
English fluency, % (n)				0.28
None	18.55% (23)	14.10% (11)	26.09% (12)	
A little	46.77% (58)	46.15% (36)	47.83% (22)	
Get by	26.61% (33)	30.77% (24)	19.57% (9)	
Well	8.06% (10)	8.97% (7)	6.52% (3)	

*EBDLS* East Bay Day Laborer Study, *PIPES* Perceived Immigration Policy Effects Scale

^a^A PIPES score of 17–34 corresponds to a mean of *never* or *rarely* responses for all 17 questions (n = 78)

^b^A PIPES score of 35–85 corresponds to a mean of more than or equal to *sometimes* responses (i.e., responded either *sometimes, often,* or *always*) for all 17 questions (n = 46)

The average PIPES score of the sample is 32.36 (SD = 12.0, range: 17–81), but 24.68 (SD = 4.84) and 45.39 (SD = 8.93) for respondents with low and high PIPES scores, respectively (Table 2). PHQ-8 and GAD-7 scores are statistically different among respondents with low and high PIPES scores. The average PHQ-8 score of the sample is 3.03 (SD = 3.48, range 0–19), and 2.05 (SD = 2.37) and 4.70 (SD = 4.36) for respondents with low and high PIPES scores, respectively. The average GAD-7 score of the sample is 3.68 (SD = 3.79, range 0–17), 2.59 (SD = 3.18) for respondents with low PIPES scores, and 5.52 (SD = 4.05) for those with high PIPES scores. Total PHQ-8 and GAD-7 scores were correlated (*r* = 0.71, p-value < 0.001). In terms of PHQ-8 depression screening, 17.74% of respondents screened positive for mild depression, 7.26% for moderate depression, and 0.81% for moderately severe depression. Respondents with high PIPES scores were more likely to screen positive for each of the three depression categories than those with low PIPES scores. In our sample, 18.6%, 7.26%, and 2.42% of respondents screened positive for mild, moderate, and moderately severe generalized anxiety disorder, respectively. Respondents with high PIPES scores were also more likely to screen positive for anxiety than those with low PIPES scores.

**Table 2:** Summary of mental health outcomes and PIPES Score among day laborers for the overall EBDLS sample and compared between low and high PIPES Scores, 2020

Variable	Total	Low PIPES^a^	High PIPES^b^	p-value
	N = 124	N = 78	N = 46	
PIPES Scores, mean (SD)	32.36 (12.03)	24.68 (4.84)	45.39 (8.93)	< 0.001
PHQ-8 Score, mean (SD)	3.03 (3.48)	2.05 (2.37)	4.70 (4.36)	< 0.001
GAD-7 Score, mean (SD)	3.68 (3.79)	2.59 (3.18)	5.52 (4.05)	< 0.001
PHQ-8 Screening, % (n)				< 0.001
Negative (0–4)	74.19% (92)	88.46% (69)	50.00% (23)	
Mild (5–9)	17.74% (22)	8.97% (7)	32.61% (15)	
Moderate (10–14)	7.26% (9)	2.56% (2)	15.22% (7)	
Moderately severe or above (15 +)	0.81% (1)	0.00% (0)	2.17% (1)	
GAD-7 screening, % (n)				< 0.001
Negative (0–4)	71.77% (89)	87.18% (68)	45.65% (21)	
Mild (5–9)	18.55% (23)	6.41% (5)	39.13% (18)	
Moderate (10–14)	7.26% (9)	5.13% (4)	10.87% (5)	
Moderately severe or above (15 +)	2.42% (3)	1.28% (1)	4.35% (2)	

EBDLS = East Bay Day Laborer Study; PIPES = Perceived Immigration Policy Effects Scale

^a^A PIPES score of 17–34 corresponds to a mean of *never* or *rarely* responses for all 17 questions (n = 78)

^b^A PIPES score of 35–85 corresponds to a mean of more than or equal to *sometimes* responses (i.e., responded either *sometimes, often,* or *always*) for all 17 questions (n = 46)

**Fig. 1:**
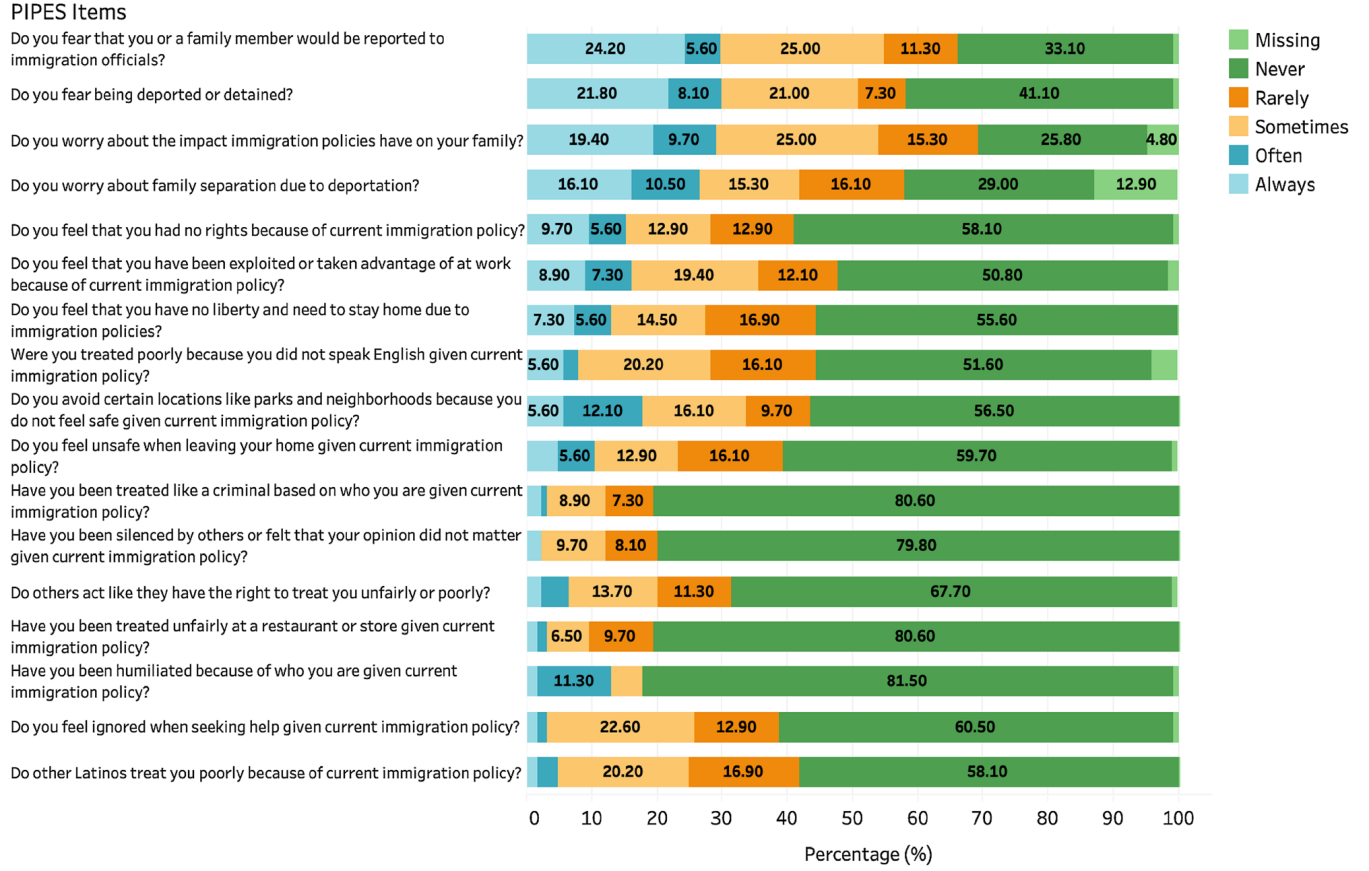
Descriptive Statistics of the 17-item Perceived Immigration Policy Effects Scale (PIPES) (n = 124), 2020. Respondents were informed of the following when beginning the matrix of PIPES items: “The next set of questions are about your experiences and feelings about current immigration policy. Please indicate how frequently you have felt the following way in your day-to-day interactions. These questions may be triggering. You can skip these questions and proceed to the next section if desired.” Color show details about Missing, Never, Rarely, Sometimes, Often, and Always response categories (Color figure online)

In Fig. 1, we display the distribution of responses for each item of the PIPES scale. Worry about family separation was the most endorsed manifestation of legal vulnerability. For example, 54.8% of respondents reported at least *sometimes* feeling concerned that they or a family member would be reported to immigration officials, and 54.1% worried about the impact that immigration policies have on their families. Likewise, 41.9% had concerns about family separation due to deportation. In terms of social exclusion, 44.3% felt they had no liberty and needed to stay home. Approximately 43.5% avoided specific locations like parks and certain neighborhoods because they did not feel safe. Similarly, 50.9% feared being deported or detained. Regarding discrimination, 47.4% of respondents had been exploited or taken advantage of at work, and 45.0% felt they had been treated poorly for not speaking English. Lastly, a minority of day laborers experienced the following manifestations of legal vulnerability: 18.0% were humiliated because of who they are, 19.4% were treated like a criminal, 19.4% were mistreated at a store or restaurant, and 20.2% were silenced by others or felt their opinions did not matter.

We removed duration in the U.S. in the final models due to its correlation with age (*r* = 0.61, p-value < 0.001). The final VIF was 1.33 for each model, indicating that multicollinearity was not of concern. As hypothesized, a higher PIPES score was associated with both depression and anxiety symptoms (Table 3). In Model 1, a one standard deviation increase in the PIPES score was associated with a 1.58 point [95% CI: 0.88, 2.29; p < 0.001] increase in PHQ-8 score controlling for all confounding variables. It is also important to note that living with a spouse is protective of depression symptoms [beta coefficient = -1.63, 95% CI: -3.05, -0.21; p < 0.05]. In Model 2, a one standard deviation increase in the PIPES score was associated with a 1.70 point [95% CI: 0.93, 2.48; p < 0.001] increase in GAD-7 score controlling for all confounding variables.

**Table 3:** Association between Perceived Immigration Policy Effects Score (PIPES) and Depression (PHQ-8) and Anxiety (GAD-7) Among Day Laborers in the East Bay, n = 124

	(1)		(2)	
	PHQ-8 Score		GAD-7 Score	
	Coeff	95% CI	Coeff	95% CI
Standardized PIPES Scores	1.583***	[0.881, 2.285]	1.704***	[0.931, 2.478]
After SIP (post 3/19/20)	0.840	[− 0.466, 2.146]	− 0.311	[− 1.750, 1.128]
Age (mean centered)	− 0.0208	[− 0.076, 0.034]	− 0.0282	[− 0.089, 0.032]
Weekly income (Ref. $0–$300)				
$301–$600	− 0.0989	[− 1.529, 1.332]	− 0.664	[− 2.240, 0.912]
$601–$1000	− 0.463	[− 2.151, 1.225]	− 1.082	[− 2.942, 0.777]
$1000 +	0.196	[− 2.296, 2.687]	0.707	[− 2.038, 3.451]
Education (mean centered)	− 0.0169	[− 0.198, 0.164]	− 0.0138	[− 0.213, 0.185]
Country of origin (Ref. Mexico)				
Guatemala	0.398	[− 1.066, 1.861]	0.475	[− 1.137, 2.088]
Other Central Am	0.570	[− 1.395, 2.535]	0.463	[− 1.702, 2.628]
Primary language (Ref. Spanish)				
Indigenous—Mam, Jakaltec	− 0.219	[− 1.828, 1.390]	− 0.0295	[− 1.803, 1.744]
Marital status (Ref. single)				
Has partner but living separately	− 0.844	[− 2.516, 0.829]	− 0.147	[− 1.989, 1.695]
Partner + living together	− 1.628**	[− 3.050, − 0.206]	− 0.951	[− 2.517, 0.616]
English ability (Ref. none or a little)	0		0	[0, 0]
Get by or well	− 0.369	[− 1.762, 1.025]	− 0.0843	[− 1.620, 1.451]
Constant	3.016***	[1.403, 4.629]	4.073***	[2.296, 5.851]
R-squared	0.228		0.210	
Adj. R-squared	0.137		0.117	
AIC	655.9		679.9	
BIC	695.4		719.4	
F	2.503		2.255	
Observations	124		124	

95% confidence intervals in brackets

* p < 0.10, ** p < 0.05, *** p < 0.010

Table 4 presents the multivariable logistic regression models analyzing the relationship between the PIPES score and a positive screening for depression and generalized anxiety. A higher PIPES score is positively associated with increased odds of a positive PHQ-8 depression and GAD-7 anxiety screening. In Model 3, a one standard deviation increase in the PIPES score was associated with 3.34 odds [95% CI: 1.80, 6.18; p < 0.001] of positive PHQ-8 screening, adjusting for control variables. In Model 4, a standard deviation increase in the PIPES score was associated with 4.43 odds [95% CI: 2.22, 8.84; p < 0.001] of positive GAD-7 screening, adjusting for control variables. Respondents who live with a spouse in the U.S. had lower odds of a positive screening for depression [OR = 0.293; 95% CI: 0.09, 1.00; p < 0.10] and anxiety [OR = 0.23; 95% CI: 0.07, 0.83; p < 0.05].

**Table 4:** Logistic regressions with outcome as positive depression screening and positive anxiety screening and Perceived Immigration Policy Effects Score (PIPES) as main predictor among day laborers in the East Bay, n = 124

	(3)		(4)	
	Positive PHQ-8 Screening		Positive GAD-7 Screening	
	OR	95% CI	OR	95% CI
Standardized PIPES Scores	3.340***	[1.804, 6.182]	4.434***	[2.224, 8.838]
After SIP (post 3/19/20)	2.155	[0.735, 6.322]	1.568	[0.540, 4.554]
Age (mean centered)	1.008	[0.966, 1.052]	0.979	[0.937, 1.023]
Weekly income (Ref. $0–$300)				
$301–$600	0.998	[0.324, 3.076]	0.848	[0.261, 2.759]
$601–$1000	0.672	[0.164, 2.749]	0.441	[0.101, 1.935]
$1000 +	1.072	[0.0954, 12.05]	3.661	[0.556, 24.12]
Education (mean centered)	0.967	[0.835, 1.119]	0.973	[0.837, 1.131]
Country of origin (Ref. Mexico)				
Guatemala	1.903	[0.543, 6.663]	1.564	[0.451, 5.428]
Other Central Am	2.781	[0.624, 12.39]	1.117	[0.227, 5.488]
Primary language (Ref. Spanish)				
Indigenous—Mam, Jakaltec	0.820	[0.226, 2.975]	1.612	[0.455, 5.702]
Marital status (Ref. Single)				
Has partner but living separately	0.664	[0.168, 2.615]	0.327	[0.0742, 1.436]
Partner + living together	0.293*	[0.0855, 1.003]	0.234**	[0.0664, 0.827]
English ability (Ref. None or A little)				
Get by or well	0.788	[0.243, 2.559]	1.031	[0.318, 3.344]
Constant	0.171	[0.044, 0.665]	0.276	[0.073, 1.044]
AIC	138.4		137.3	
BIC	177.9		176.8	
Observations	124		124	

Exponentiated coefficients; 95% confidence intervals in brackets

*p < 0.10, **p < 0.05, ***p < 0.010

## Discussion

We examined whether legal vulnerability resulting from exclusionary immigration policies was associated with an increased risk of poor mental health outcomes among Latino day laborers. Consistent with our hypothesis, our findings suggest that legal vulnerability is associated with more depressive and anxiety symptoms among this group. Among our sample, broader anti-immigrant social and political contexts are consequential for respondents’ mental health. Despite living in California, a sanctuary state, immigration policy vulnerability is associated with unfavorable mental health outcomes for immigrants. As a structural determinant of health, exclusionary immigration policy contributes to health inequities. We posit that the current socio-political environment exacerbates mental health outcomes among day laborers through unhealthy behavioral coping strategies and increased stress exposure. For instance, as a defense mechanism to anticipated and experienced discriminatory events, day laborers may turn to concealment and social isolation, which are associated with adverse mental health [4]. In terms of gender role expectations, unemployment and financial hardship result in male day laborers avoiding communication with family back home due to failing to be adequate breadwinners, thereby exacerbating social isolation and stress [48]. Simultaneously, the excessive distress attributed to discriminatory encounters, the perpetual fear of family separation (for those with family in the U.S.), and the overall precarious experiences while looking for work may lead to the “wear and tear” of day laborers’ mental health, and ultimately, their physical health [16, 49]. The cumulative stressors they experience may repeatedly trigger the stress-response system and lead to high allostatic load, which leads to the deterioration in the functioning of the cardiovascular, metabolic, endocrine, cognitive, and immune systems [50].

Steps to mitigate immigration policy vulnerability, such as a pathway to citizenship or employment authorization, can prevent anxiety and depression symptoms among Latino day laborers. The last congressionally supported amnesty in the U.S. was the 1986 Immigrant Reform and Control Act (IRCA), which established a legalization program for almost 3 million undocumented immigrants; simultaneously, IRCA criminalized the hiring of undocumented workers and increased resources for border enforcement [51]. Thirty-five years after IRCA’s passage, no legislative path to citizenship has occurred, and 11 million undocumented individuals live in limbo in the U.S. with limited employment opportunities. Current proposals that can partially address this include the 2021 Farm Workforce Modernization Act (FWMA), which would create a green card option for long-term agricultural workers and a new temporary worker visa program [52]. A more comprehensive path to citizenship than FWMA, however, would still be needed. Developing mental health interventions to help this group of migrant workers should also be a high policy priority. Day laborers have limited access to preventive health care, let alone behavioral health. Consequently, it is vital to create more timely and accessible mental health interventions that overcome access and language barriers, such as digital health interventions [53]. Moreover, the experiences of day laborers and other migrant workers during the COVID-19 pandemic are likely to worsen due to the lack of access to federal programs to help families weather the pandemic's economic effects.

The study has limitations to consider when interpreting the findings. First, we rely on day laborers’ self-reported health measures rather than independent clinical diagnoses by health care professionals. However, the well-validated instruments used to detect depression and anxiety are commonly used in migrant and ethnic populations and clinical settings [46]. Second, as a form of self-protection, some respondents may have minimized their experiences of discrimination and amplified their perceptions of personal control over their living situations, which would underestimate the detected effect of immigration policy vulnerability on mental health. For instance, day laborers may not report the true extent to which they experience discrimination while looking for work or may underreport their depression and anxiety symptoms. Third, our data is cross-sectional, and temporality between the immigration policy vulnerability and mental health outcomes cannot be established. Fourth, our study's findings cannot be extrapolated to outside of California, a sanctuary state where the context of reception is relatively more inclusive of immigrants. Last, we did not assess individual experiences of discrimination not attributed to immigration policy. Other forms of cultural, interpersonal, and internalized discrimination can also lead to adverse mental health outcomes and are potential unmeasured confounders. Nonetheless, even if interpersonal discrimination were entirely eradicated or accounted for in our model, health inequities would likely persist due to structural racism in immigration policies [15, 54].

Future work is needed to fully understand the multilevel forms of discrimination that affect day laborers’ mental health outcomes across the life course. Acknowledging that discrimination is produced and maintained at multiple levels [15, 54], future studies should differentiate between internal, interpersonal, and institutional sources of discrimination. Doing so will help determine where and how to intervene to prevent the exacerbation of health disparities for precarious migrant workers. Furthermore, given that immigration policy has spillover effects, future work should focus on the impacts of legal vulnerability among heterogeneous groups of immigrants and their networks. Beyond politically supportive environments, future studies can also focus on new settlement destinations and overly restrictive states, including Texas, Alabama, and Georgia, to improve our understanding of how variation in immigration policy vulnerability affects day laborers’ mental health. Finally, we recommend continuing empirical research on the risk and protective factors associated with mental health among Latino men in the US.

## Contributions to the Literature

While previous studies have examined the effects of abuse and deprivation on day laborers’ health, no study has linked day laborers’ experiences of immigration policy vulnerability and mental health outcomes. Our work contributes to the evidence of the association between exclusionary political environments and adverse mental health outcomes among immigrants. Furthermore, this is one of the first studies to operationalize legal vulnerability as a multidimensional construct comprised of fear of family separation, social exclusion, and discrimination. These findings have important implications for understanding how legal vulnerability manifests in the everyday lives of immigrants.
